# Effect of Cosolvent on the Vesicle Formation Pathways under Solvent Exchange Process: A Dissipative Particle Dynamics Simulation

**DOI:** 10.3390/molecules28135113

**Published:** 2023-06-29

**Authors:** Zhonglin Luo, Zhou Shu, Yi Jiang, Biaobing Wang

**Affiliations:** Jiangsu Key Laboratory of Environmentally Friendly Polymeric Materials, Jiangsu Collaborative Innovation Center of Photovolatic Science and Engineering, National Experimental Demonstration Center for Materials Science and Engineering, School of Materials Science and Engineering, Changzhou University, Changzhou 213164, China; zhoushu202306@163.com (Z.S.); yijiang202306@163.com (Y.J.)

**Keywords:** cosolvent, solvent exchange self-assembly, liquid–liquid phase separation, vesicle formation mechanism, amphiphilic diblock copolymer, dissipative particle dynamics (DPD) simulation

## Abstract

The effective control over the vesicle formation pathways is vital for tuning its function. Recently, a liquid–liquid phase-separated intermediate (LLPS) is observed before a vesicular structure during the solvent exchange self-assembly of block copolymers. Though the understanding of polymer structures and chemical compositions on the competition between LLPS and micellization has made some progress, little is known about the role of cosolvent on it. In this study, the influence of cosolvent on the vesicle formation pathways is investigated by using dissipative particle dynamics. The results show that the range of water fraction within which the LLPS is favored will be highly dependent on the affinity difference of cosolvent to water and to polymer repeat units. The change of the cosolvent–water interaction and the water fraction impact the distribution of cosolvent in the polymer domain, the miscibility between the components in the system as well as the chain conformations, which finally induce different self-assembly behaviors. Our findings would be helpful for understanding the LLPS and controlling the morphologies of diblock polymers in solutions for further applications.

## 1. Introduction

The amphiphilic block copolymers form various structures in solution, such as spherical micelles, cylindrical micelles, vesicles, and so on [1,2]. Among them, the vesicular structures have attracted special and continuous attentions in the last few decades because of their great potential applications of drug delivery [3,4,5], nanoreactors [6], mimics of cells or organelles [7,8], and so on [9]. For achieving these functions, the rigorous control over the morphology evolution of the vesicle is critical. With this in mind, scientists from various backgrounds have make great efforts to investigate the self-assembly behaviors of diverse systems and to explore the physicochemical principles behind them.

Despite many documents focused on the subject, it has been generally recognized that there are only two fundamental ways for vesicle formation described as Mechanism I (by bilayer-closing) [10] and Mechanism II (via semi-vesicle intermediate) [11,12]. In the former pathway, the bilayer membrane structures, such as rod-like, disk-like, or sheet-like intermediates are observed first and then the bilayer membranes bend and close to form the vesicles. This mechanism is frequently reported in experimental and theoretical studies [13,14,15]. In the second pathway, a spherical semi-vesicle intermediate with a small hollow cavity is formed by the diffusion of hydrophilic blocks as well as solvent molecules towards the center of the spherical micelle, and then the full vesicle grows up gradually. This mechanism is initially proposed by using a field-based simulation method, and also observed by some experimental studies [16]. It is usually agreed that the second pathway is preferential when the polymer concentration is much lower and the hydrophobic property of the block copolymer is higher [12,17].

Recently, based on the observation of the phase separation of polymer-rich liquid droplets by time-resolved in situ monitoring technique and the self-consistent mean field (SCF) theory, another mechanism is proposed that the vesicle evolves from the growth and internal phase separation of a polymer-rich liquid droplet precursor in the liquid–liquid phase-separated state [18]. It is thought that in a liquid mixture with a specific composition of cosolvent (good solvent for all blocks) and water, the polymer-rich liquid droplet (LL) is more thermodynamically favorable than dissolution or self-assembly [19,20]. Though the LL is also reproduced in simulations [21,22], as we know, it still lacks the direct observation of how the phase separation within the LL develops into a full vesicular structure by particle simulations. Moreover, most works are concerned about the influences of polymer structures and chemical compositions on the formation and stability of the liquid–liquid phase separation (LLPS) [19,23], but no research examines the role of the cosolvent during this process.

For a long period of time, the observation of a self-assembly process of amphiphilic block copolymers in simulations is often performed by using a single-component solvent which is supposed as having the averaged property of the binary mixture [24,25,26]. However, experimental evidence suggests that the choice of different cosolvents influences nanoparticle sizes [27,28] as well as structures [29,30], the cosolvent/selective solvent ratio impacts chain conformation [31,32], and the distribution of the cosolvent in the polymer domain is distinguished from that in the bulk solution [33]; moreover, the cosolvent dispersed in the core and the corona also may be much different [29,30,32]. All of the phenomena indicate the important role of the cosolvent in the self-assembly process and should be considered individually [21,34,35].

In this study, dissipative particle dynamic (DPD) simulation is used to investigate the vesicle formation process of the model amphiphilic diblock copolymer under solvent exchange. We show that the distribution of cosolvent and water in the polymer domain is strongly dependent on the affinity difference of cosolvent to water and to the polymer segments, which further impact the chain conformation and induce different self-assembly behaviors. Moreover, a cosolvent-dependent critical water fraction is found for the observation of the LLPS. Our findings would be helpful for the efficient engineering of nanostructures.

## 2. Results and Discussion

### 2.1. Direct Self-Assembly of Diblock Copolymer in Water

**Figure 1:**
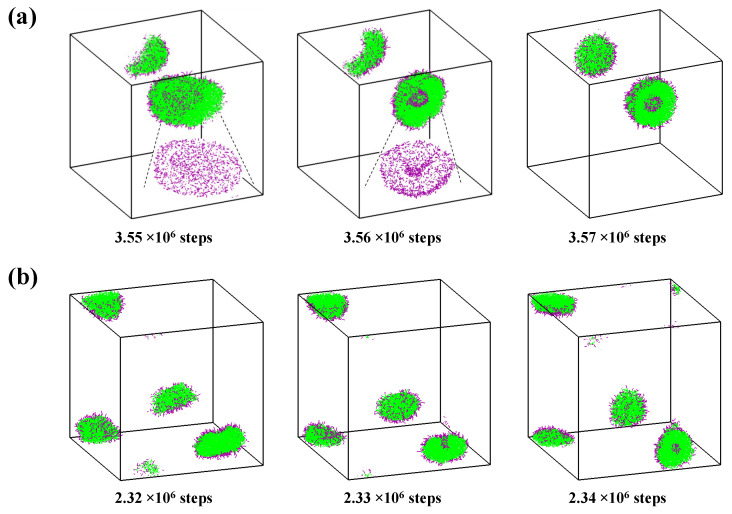
Two trajectories of the direct self-assembly of A_2_B_12_ in water. Insets correspond to the integral morphologies of aggregates. Insets depict the integral disk-like and bowl-like intermediates. Hydrophilic and hydrophobic segments are in purple and green, respectively. For clarity, water is not shown and only hydrophilic segments are shown in the insets.

For comparison with the self-assembly behaviors under solvent exchange, the self-assembly of A_2_B_12_ starting from a homogeneous dispersion in pure water is also performed. The typical snapshots as a function of time in two simulation trajectories are shown in Figure 1. In the first trajectory, all chains are aggregated into a disk-like micelle and then a vesicle is formed by bending and closing the bilayer membrane (Figure 1a). The insets clearly show the integral disk-like and bowl-like intermediates. In the second trajectory, a small disk-like structure is observed at the earlier time. To compare with the first trajectory quantitatively, the size of the clusters is characterized by the number of aggregated chains in it (*n*_agg_), and the clusters are identified by the contacts between the hydrophobic segments with a distance less than 1.5 *r*_cut_. Here, the small disk-like structure has *n*_agg_ of 511, and it forms a vesicle quickly via the local collapse of the surface (Figure 1b). Clearly, both trajectories follow the “bilayer-closing” mechanism (Mechanism I) [10]. It is reported that vesicles also could form via a semi-vesicle pathway (Mechanism II) [12]. We did not observe this process in our A_2_B_12_ system from self-assembly in water; it may be the relative moderate hydrophobic property of *a*_BW_ = 50 adopted in this study [17].

### 2.2. Self-Assembly in Liquid Mixture Where Cosolvent and Water Is Attractive

**Figure 2:**
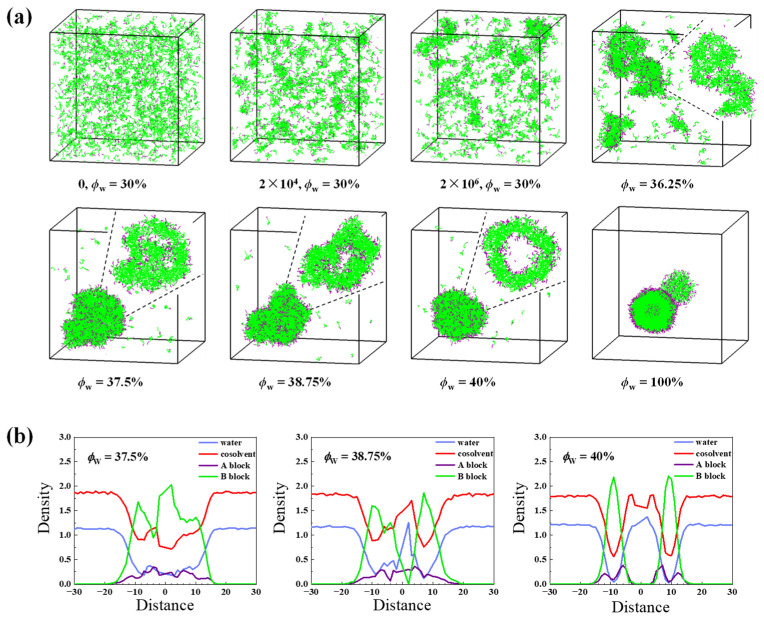
(**a**) Typical snapshots of the vesicle formation process during solvent exchange in the case of *a*_WG_ = 15 starting from $\phi_{W}^{\mathrm{init}}$ = 30%. Insets correspond to the section views of aggregates; (**b**) Density profiles of water, cosolvent, and copolymer blocks versus the distance from the mass center of the corresponding morphologies in (**a**). Water, cosolvent, hydrophilic, and hydrophobic segments are in blue, red, purple, and green, respectively. For clarity, the solvents are not shown in the snapshots.

In the case of *a*_WG_ = 15, the difference between *a*_WG_ and *a*_AG_ (*a*_BG_) is −10, which means the cosolvent and water is highly attractive. As it is seen in Figure 2a, when the self-assembly starts from $\phi_{W}^{\mathrm{init}}$ = 30%, the randomly dispersed chains gather into many small clusters (the *n*_agg_ of the largest cluster is 161, the second 90, and others 16~34) after 2 × 10^6^ time steps for equilibrium. With the increase of *ϕ*_W_, small clusters merge continuously and at *ϕ*_W_ = 36.25%, two big irregular clusters are seen in the simulation box. The section view of the biggest one (seen in the insertion) demonstrates that the hydrophilic and hydrophobic blocks are not segregated. These disordered clusters indicate LLPS [18] and are also observed by other simulations [21,22]. Then a large irregular cluster is formed at *ϕ*_W_ = 37.5%, and the section slide shows several cavities exist in the interior and that the outer hydrophobic membrane has been basically formed. Afterwards, the discrete cavities merge with each other at *ϕ*_W_ = 38.75% and finally, a most matured vesicle is found at *ϕ*_W_ = 40%.

The morphologies at *ϕ*_W_ = 37.5%, 38.75%, and 40% are further characterized by analyzing the profiles of number density of all components (water, cosolvent, block A, and block B) along the long axis of the clusters, respectively (Figure 2b). At *ϕ*_W_ = 37.5%, blocks A and B are almost randomly distributed in the cluster containing a large amount of solvents because of the irregular shapes and dispersion of cavities. It also indicates that the hydrophilic and hydrophobic blocks are not clearly segregated. At *ϕ*_W_ = 38.75%, hydrophobic blocks move away from the center. At *ϕ*_W_ = 40%, the hydrophobic membrane and the interior/exterior shells are clearly seen. Moreover, the solvent molecules are going away from the hydrophobic membrane simultaneously. Therefore, the density profiles shown in Figure 2b further prove the phase separation process (rearrangement) within the polymer-rich liquid droplet. It is noteworthy that, besides the phase separation (or internal rearragment) in the cluster, the size of the cluster shrinks gradually as indicated by the decreased distance of blocks A and B from the center of the aggregates. The formation of LLPS and the subsequent rearrangement is also observed by a long simulation of 4.0 × 10^6^ time steps at a fixed *ϕ*_W_ = 40% (Figure S1).

**Figure 3:**
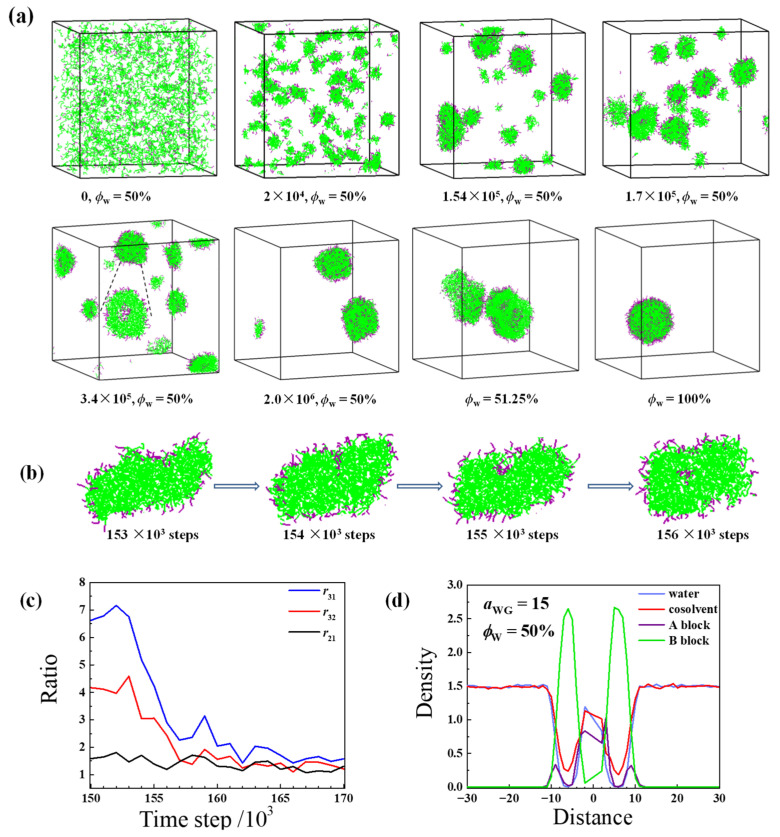
(**a**) Typical snapshots of the vesicle formation process observed during solvent exchange in the case of *a*_WG_ = 15 starting from $\phi_{W}^{\mathrm{init}}$ = 50%. The inset corresponds to the section view of a vesicle; (**b**) The semi-vesicle formation during the earlier stage of (**a**). For a better understanding, a section of one elongated semi-vesicle is given; (**c**) The ratios between the three eigenvalues of the radius of the gyration tensor as a function of time for the elongated semi-vesicle shown in (**b**); (**d**) Density profiles of water, cosolvent, and copolymer blocks versus the distance from the mass center of the largest vesicle formed at 3.4 × 10^5^ time steps and *ϕ*_W_ = 50%. The colors of water, cosolvent, hydrophilic, and hydrophobic segments are in blue, red, purple, and green, respectively. For clarity, the solvents are not shown in the snapshots.

Strikingly, when the self-assembly starts from the solvent composition $\phi_{W}^{\mathrm{init}}$ = 50%, the vesicle formation undergoes a different way. As shown in Figure 3a, during 2.0 × 10^6^ time steps of equilibrium at *ϕ*_W_ = 50%, the randomly dispersed chains are quickly aggregated; tiny irregular micelles, small cylindrical structures, and small vesicles are observed successively, and finally, two vesicles are observed. Afterwards, with the further increase of *ϕ*_W_, two vesicles fuse into a large one. Careful analysis indicates that some small elongated vesicles are present at the very earlier stage of the self-assembly process. For example, Figure 3b shows the evolution of the largest aggregate (*n*_agg_ = 267) during 1.53 × 10^5^ to 1.57 × 10^5^ time steps. It is seen that an elongated vesicle is formed by diffusion of the hydrophilic blocks into the center of the small cylinder.

In order to describe the instantaneous shape of the aggregates, we calculate the three eigenvalues λ_1_, λ_2_, and λ_3_ of the radius of the gyration tensor, which is defined as [34]:

(1) $$A=\left[\begin{array}{ccc} S_{xx} & S_{xy} & S_{xz}\\ S_{yx} & S_{yy} & S_{yz}\\ S_{zx} & S_{zy} & S_{zz} \end{array}\right]$$

and:

(2) $$S_{ab}=\frac{1}{n}\sum_{i=1}^{n}\left(a_{i}-a_{\mathrm{cm}}\right)\left(b_{i}-b_{\mathrm{cm}}\right)$$

where *a* and *b* denote *x*, *y*, or *z* components of the bead’s coordinates, *a_i_* and *a*_cm_ for the *i*th-bead and center-of-mass of the aggregate, respectively, and *n* is the total number of beads in the aggregate. In the following, we sort the three eigenvalues of the matrix of Equation (3) as *λ*_1_ ≤ *λ*_2_ ≤ *λ*_3_. It is expected that *R*_g_^2^ = *λ*_1_ + *λ*_2_ + *λ*_3_. Then, the ratios of the three eigenvalues are presented by *r*_31_ =*λ*_3_/*λ*_1_, *r*_32_ =*λ*_3_/*λ*_2_, and *r*_21_ =*λ*_2_/*λ*_1_, respectively. Here, *r*_31_ is the ratio of the long axis to the short axis, and the *r*_21_ is the ratio of the long axis to the middle axis. The larger the value of the two, the closer the shape of the aggregate is to the cylinder-like; *r*_32_ is the ratio of the middle axis to the short axis. Its value is close to 1 and indicates that the cross-section of the aggregate is circular.

As shown in Figure 3c, at 1.5 × 10^5^ time steps, *r*_31_ is much larger than *r*_32_ and *r*_21_, and *r*_21_ is around 1.5. Therefore, the aggregate is flat cylinder-like with a long axis. With the time increases, *r*_31_ and *r*_32_ decrease quickly and the three ratios become very close after 1.62 × 10^5^ time steps, indicating the subsequent structural transformation from the elongated semi-vesicle into a spherical vesicle. This process is very similar to an in-between pathway between Mechanism I (by bilayer-closing) and Mechanism II (via semi-vesicle intermediate) reported in the literature [17]. The density profile of the largest aggregate at 3.4 × 10^5^ time steps is shown in Figure 3d. We can see that the vesicle is well organized and the hydrophobic membane is dense with only a few of the cosolvent beads within it.

**Figure 4:**
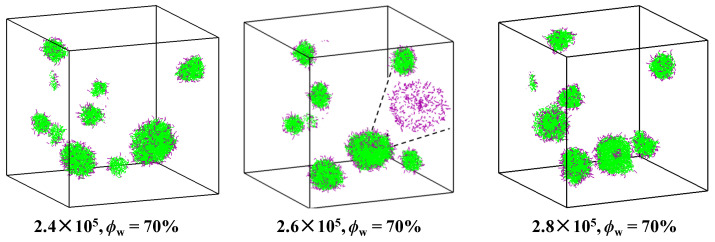
Typical snapshots of the vesicle formation observed at the equilibrium stage of $\phi_{W}^{\mathrm{init}}$ = 70% in the case of *a*_WG_ = 15. The colors of hydrophilic and hydrophobic segments are in purple and green, respectively. For clarity, solvents are not shown and only hydrophilic segments are shown in the inset.

When the self-assembly starts from $\phi_{W}^{\mathrm{init}}$ = 70%, a typical dense disk-like intermediate with *n*_agg_ = 476 is found at 2.4 × 10^5^ time steps, as shown in Figure 4. After the local collapse of the disk surface at 2.6 × 10^5^ time steps (seen in the inset of Figure 4), a small vesicle is seen. This process undergoes very quickly at *ϕ*_W_ = 70%. To verify the self-assembly behavior, five independent simulations are carried out. Small and dense vesicles are formed first by the local collapse of the disk surface in all five simulations, and the average *n*_agg_ of the first observed vesicles is 407 ± 52.

### 2.3. Self-Assembly in Liquid Mixture Where Cosolvent and Water Is Repulsive

**Figure 5:**
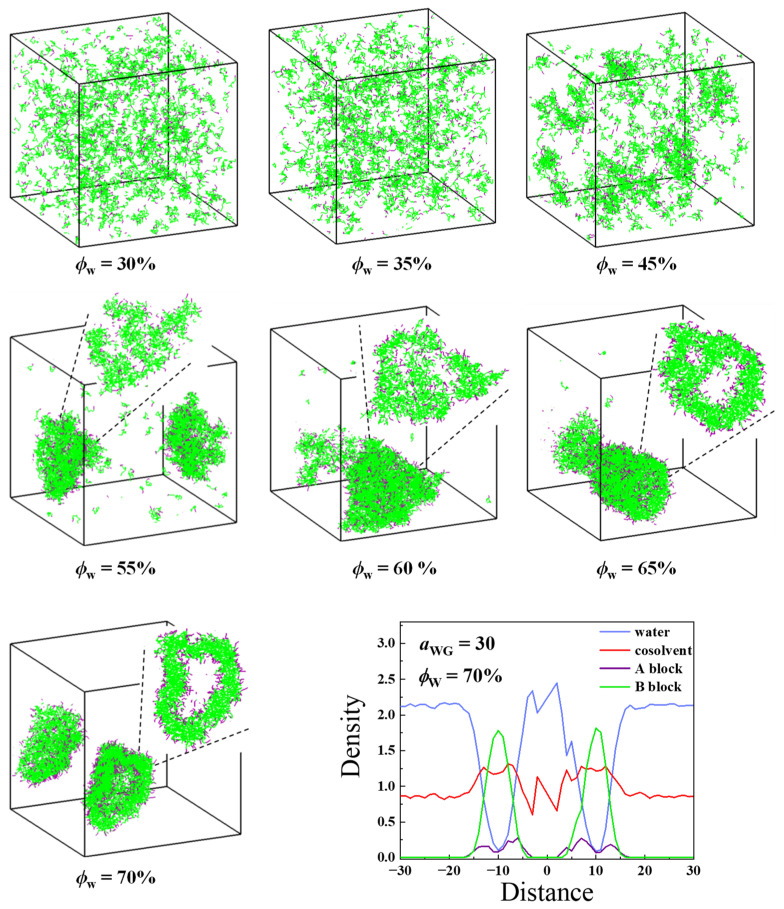
Typical snapshots of the vesicle formation process observed during solvent exchange in the case of *a*_WG_ = 30 starting from $\phi_{W}^{\mathrm{init}}$ = 30% and the density profile of the structure observed at *ϕ*_W_ = 70%. Insets correspond to the section views of aggregates. The colors of water, cosolvent, hydrophilic, and hydrophobic segments are in blue, red, purple, and green, respectively. For clarity, the solvents are not shown in the snapshots.

In the case of *a*_WG_ = 30, the difference between *a*_WG_ and *a*_AG_ (*a*_BG_) is 5, which means the cosolvent and water is repulsive but marginally misicible. The self-assembly of A_2_B_12_ starting from $\phi_{W}^{\mathrm{init}}$ = 30% is shown in Figure 5. After equilibrium for 2.0 × 10^6^ time steps, only very tiny aggegates are seen, which means the polymer chains are more soluble when the cosolvent and water is weakly repulsive. Therefore, a slow exchange frequency of *t*_eq_ = 6 × 10^5^ time steps is adopted at each solvent switch step for cluster coalescence.

As shown in Figure 5, most chains are free in the solvent mixture at *ϕ*_W_ = 30%. With the increase of *ϕ*_W_, some small cottony aggregates with *n*_agg_ = 20~32 are formed at *ϕ*_W_ = 35%. The snapshot and density profile of the largest cluster (*n*_agg_ = 32) indicate that those small cottony aggregates are LLPS intermediates (Figure S2). These aggregates fuse continuously and at *ϕ*_W_ = 55%, most polymer chains are involved into a big irregular cluster. The section slide of the cluster indicates that the polymer chains are loosely agglomerated and the hydrophilic and hydrophobic blocks are not segregated. Afterwards, phase separation in the cluster takes place gradually, the closed aqueous cavities form and join together, and finally, a most matured vesicle is seen at *ϕ*_W_ = 70%. Therefore, the self-assembly progress starting from $\phi_{W}^{\mathrm{init}}$ = 30% in the case of *a*_WG_ = 30 is similar to that in the case of *a*_WG_ = 15, but the evolution from LLPS to vescile occurs later and the LLPS could be observed starting from a broader $\phi_{W}^{\mathrm{init}}$ range. Moreover, lots of cosolvents adsorbed on the vesicle membrane, and the membrane is wide and loose, as demonstrated by the density profile analysis.

The self-assembly of A_2_B_12_ starting from $\phi_{W}^{\mathrm{init}}$ = 60% is shown in Figure S3; with the time increases, cottony structures composed of loosely gathered micro-domains are seen during the equilibrium process. They develop into a large quasi-spherical aggregate at 2.0 × 10^6^ time steps without obvious segregated hydrophobic and hydrophilic domains. With the cosolvent gradually exchanged to water, internal phase separation continues and finally, a most matured vesicle is seen at *ϕ*_W_ = 70%. Afterwards, the vesicle membrane becomes dense, and the vesicle and the internal cavity shrink gradually. Therefore, the vesicle formation also obeys the phase separation mechanism.

**Figure 6:**
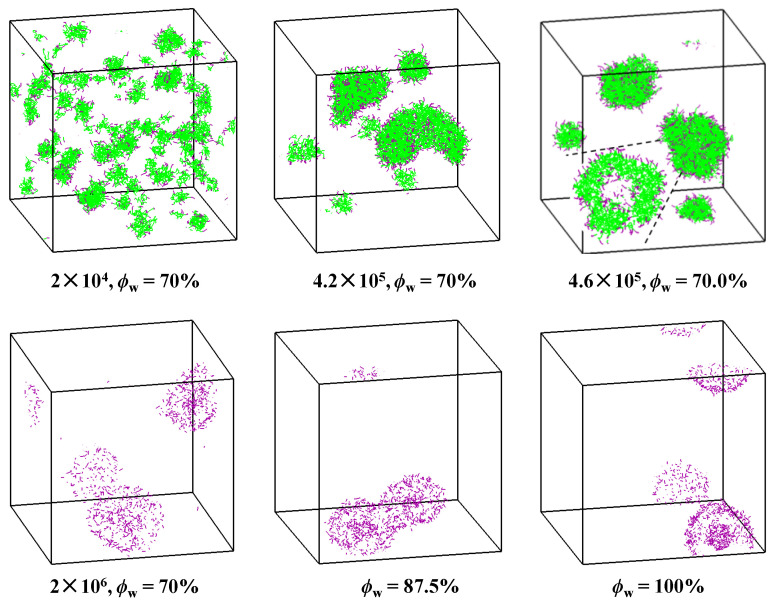
Typical snapshots of the vesicle formation process observed during solvent exchange in the case of *a*_WG_ = 30 starting from $\phi_{W}^{\mathrm{init}}$ = 70%. The inset corresponds to the section view of the aggregate. The colors of hydrophilic and hydrophobic segments are in purple and green, respectively. For clarity, the solvents are not shown in the snapshots and only hydrophilic segments are shown for vesicular structures.

When $\phi_{W}^{\mathrm{init}}$ is 70%, though some small aggregates with local collapses on the surface are found at a very early stage, they could not develop into spherical vesicles because of the small sizes (with *n*_agg_ = 200~250). When time increases, a sheet-like intermediate with *n*_agg_ = 483 is seen at 4.2 × 10^5^ time steps after mergence of the small aggregates, which then bends and closes into a vesicle at 4.6 × 10^5^ time steps (Figure 6). Compared with the vesicle observed in Figure 4, the formed structure is highly swelling and the apparent size is much larger. Finally, two vesicles are observed after 2 × 10^6^ equilibrium time steps. They fuse into a big one with the further increase of *ϕ*_W_. Therefore, when $\phi_{W}^{\mathrm{init}}$ = 70%, the vesicle formation obeys a typical Mechanism I pathway. We also ran five simulations starting from $\phi_{W}^{\mathrm{init}}$ = 70%, the average *n*_agg_ of the first observed vesicles is 426 ± 54, which is a bit less than the value in the case of *a*_WG_ = 15. However, the apparent sizes of these vesicles are much larger. Moreover, all trajectories show the typical bilayer bending process.

### 2.4. The Physiochemical Principles behind the Different Self-Assembly Behaviors

**Figure 7:**
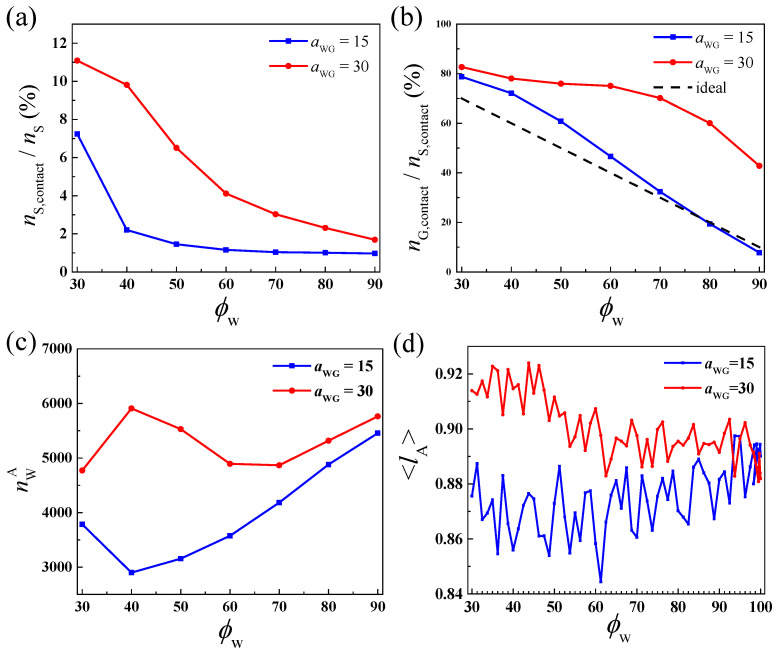
(**a**) The percentage of solvents which contact with polymer; (**b**) The percentage of cosolvent in the contacted solvents; (**c**) The number of water beads which contact with block A, and (**d**) The average bond length of block A as a function of *ϕ*_W_ in the case of *a*_WG_ = 15 and *a*_WG_ = 30.

To understand the phys-chemical basis behind the behaviors in the different solvent mixtures, the fractions of solvent beads (both cosolvent and water) which contact with polymers (distance to polymer beads *r* ≤ 1.0) and the percentages of the cosolvent beads in the contacted solvent beads are shown in Figure 7a,b. Both values are higher in the case of *a*_WG_ = 30, which indicates that the cosolvent is preferentially adsorbed in the polymer phase when the cosolvent and water are weakly repulsive. The high cosolvent content in the clusters would weaken the repulsion interactions of *a*_AB_ and *a*_BW_. Therefore, when *a*_WG_ is higher, there are more free chains in the system, the LLPS could be observed starting from a broader $\phi_{W}^{\mathrm{init}}$ range, and the formed clusters swell highly. Moreover, in the case of *a*_WG_ = 15, the percentage of cosolvent in the contacted solvents is higher than the average *ϕ*_G_ (= 1 − *ϕ*_W_) if *ϕ*_W_ < 80%, which means that the cosolvent is also slightly enriched in the polymer phase even in a strongly attractive liquid mixture.

The number of water beads which contact with block A ($n_{W}^{A}$) is further calculated. Interestly, in the case of *a*_WG_ = 15, $n_{W}^{A}$ shows the lowest value at about *ϕ*_W_ = 40% (Figure 7c), and the values at *ϕ*_W_ = 40% and 50% are obviously less than that at *ϕ*_W_ = 30%. It means the addition of water, a second good solvent to hydrophobic blocks, induces the depletion of both solvents. Therefore, the hydrophilic blocks collapse in the liquid mixture composed of two good solvents, which is called the cononsolvency phenomenon [36,37,38]. As shown in Figure 7d, the average bond length of hydrophilic blocks shrinks in the case of *a*_WG_ = 15 while it is stretched in the case of *a*_WG_ = 30 when *ϕ*_W_ < 60%. The hydrophobic blocks agglomerate quickly in the former to avoid exposing to water. The collapsed hydrophilic chains also indicate that the high affinity between cosolvent and water would promote the immiscibility of the copolymer chains in the solvent mixture, thus, the apparent hydrophobic property of the copolymers seems improved and it explains why the small elongated semi-vesicles are observed before vesicle formation at *ϕ*_W_ = 50%.

## 3. Methods and Parameters

### 3.1. DPD Simulations

DPD is a mesoscale method for the simulations of coarse-grained systems over long length and time scales. In DPD, a bead having mass *m* represents a block or a cluster of atoms or molecules moving together in a coherent fashion. In this study, the amphiphilic diblock copolymers are simplified into linear coarse-grained chains composed of hydrophilic A beads and hydrophobic B beads. A model diblock copolymer A_2_B_12_ with a short hydrophilic segment is constructed as it is reported that LLPS is preferred over micellization at a weak hydrophilicity of the block copolymer, e.g., 25% or less [18,20]. Two types of solvent beads, beads W which present a selective solvent for A beads (e.g., water) and beads G which means a good solvent for both A and B beads, are considered in the systems. The forces acting on a bead *i* can be described by:

(3) $$f_{i}=\sum_{j\neq i}\left(F_{ij}^{C}+F_{ij}^{D}+F_{ij}^{R}\right)+f_{i}^{S}$$

where $F_{ij}^{C}$ is a soft conservative repulsive force, $F_{ij}^{D}$ is a dissipative force for viscous drag, and $F_{ij}^{R}$ is a stochastic impulse force [39]. All three forces which act over each bead are within a cutoff radius *r*_cut_, beyond which the forces vanish [40]. Specifically, the conservative force $F_{ij}^{C}$ is expressed as:

(4) $$F_{ij}^{C}=\left\{\begin{array}{lll} a_{ij}(1-r_{ij}/r_{\mathrm{cut}}) & & \left(r_{ij}\;<\;r_{\mathrm{cut}}\right)\\ 0 & & \;(r_{ij}\;>\;r_{\mathrm{cut}}) \end{array}\right.$$

where *a_ij_* denotes repulsive parameters between two beads in $F_{ij}^{C}$ and is given in Table 1.

We set *a_ii_* = *a_jj_* = 25 for any two beads of the same kind. As found by Groot and Warren, ∆*a_ij_* = *a_ij_* − *a_ii_* is proportional to the Flory–Huggins parameter χ*_ij_* [40]. Specifically, for the system with the reduced number density ρ = 3, χ*_ij_* = (0.286 ± 0.002) × ∆*a_ij_*. Therefore, *a*_AG_ = *a*_BG_ = 25 indicates G is a cosolvent (good solvent) for A and B, while *a*_AW_ = 25 and *a*_BW_ = 50 mean W is a selective solvent to A. The strong repulsion *a*_AB_ = 50 represents that beads A and B are immiscible, which are ubiquitous for polymer components. The solvent–cosolvent interactions *a*_WG_ is set to 15 or 30. Specifically, *a*_WG_ < 25 presents an attractive liquid mixture, for example, the dimethyl sulfoxide and water mixture exhibits a negative free energy of mixing throughout the concentration range because of the strong hydrogen bonds [41]. The value of *a*_WG_ = 30, whose corresponding χ_WG_ = 0.286 × (30 − 25) = 1.43 is below the critical value for phase separation of monomer blends (χ^crit^ = 2.0), means the solvent and cosolvent are marginally miscible, for example, the THF and water mixture [42]. It is worth noting that it is the difference between *a*_WG_ and *a*_AG_ (=*a*_BG_) which determines the relative miscibility among cosolvents, water, and copolymers [34]. For the A_2_B_12_ copolymer, the vesicular structure is thermodynamically stable in the final polymer solution (see below). Our preliminary results indicate that the solvent exchange self-assembly behaviors between *a*_WG_ = 15~30 are in the middle of the two cases. Therefore, in this study, we only discuss the self-assembly processes of A_2_B_12_ copolymers under the two extreme and typical instances.

For adjacent bonded beads, a harmonic spring force $f_{i}^{S}=\sum_{j}C^{S}r_{ij}$ is used, where *C*^S^ is the spring constant. To keep the balance of the accuracy and the efficiency of DPD simulations, the mass, length, and energy presented by *m*, *r*_cut_, and *k*_B_*T*, respectively, are set to *m* = *r*_cut_ = *k*_B_*T* = 1, where *k*_B_ is the Boltzmann constant, *T* is the temperature, and the time unit τ = *r*_cut_ (*m*/*k*_B_*T*)^1/2^ = 1. The magnitude of spring constant *C*^S^ and the dissipation parameter are chosen to be 4.0 and 4.5, respectively. The time step is ∆*t* = 0.05τ.

### 3.2. Solvent Exchange Method

All the simulations are performed in a 60 × 60 × 60 periodic box containing 6.48 × 10^5^ beads (ρ = 3). Initially, 1000 coarse-grained chains of A_2_B_12_, corresponding to the volume fraction of copolymers (*f*_p_) about 2.16% are dispersed randomly in a mixed solvent composed of W and G beads. The interaction parameters *a_ij_* for all bead pairs are set to 25, and after 1.0 × 10^5^ time steps for equilibrium, a homogenous mixture is obtained. Then, the pair interaction parameters shown in Table 1 are imposed to the beads and after 2.0 × 10^6^ time steps of equilibrium, the self-assembly process is monitored by using a solvent exchange strategy to mimic the dialysis process in the experiment.

Especially, in each solvent exchange step, the number of exchanged G beads (*n*_G,ex_) is determined by the bead number that is supposed to switch (*n*_G,sup_) and the number of beads outside of the polymer aggregates or away from single chains (*n*_G,out_). If *n*_G,out_ > 2 × *n*_G,sup_, *n*_G,ex_ is equal to *n*_G,sup_, otherwise, *n*_G,ex_ is set to 0.5 × *n*_G,out_. After each solvent switch, the system is equilibrated with a time duration of *t*_eq_, then, the next step is performed until less than 200 G beads are left in the system. Finally, the trace amount of G beads is switched and there are only W beads present in the systems.

The relative content of W beads in the mixed solvent (*ϕ*_W_), defined as [42] *n*_W_/(*n*_W_ + *n*_G_) × 100%, is used to describe the solvent composition. Here, *n*_W_ and *n*_G_ are the number of W beads and G beads in the simulation box, respectively. The initial water fraction *ϕ*_W_ ($\phi_{W}^{\mathrm{init}}$) is set to 30% because below that concentration, no featured aggregates are found (see the Section 2 Results and Discussion). The supposed exchange quantity at each step *n*_G,sup_ is equivalent to 1.25% of the total solvent beads, and *t*_eq_ is 2.0 × 10^5^ time steps. As observed in experiments, the morphology of the aggregates could be kinetically trapped by the cosolvent/water ratio; therefore, different $\phi_{W}^{\mathrm{init}}$ values are also adopted to investigate the self-assembly process. All the DPD simulations were conducted using DL_MESO 2.4 software [43].

## 4. Conclusions

In this study, the influence of cosolvent on the vesicle formation pathways is investigated by using dissipative particle dynamics. It is found that the presence of LLPS will be highly dependent on the affinity difference of cosolvent to water and to polymer repeat units. If the cosolvent and water is strongly attractive, LLPS occurs at the low water fraction, the segregation of polymer segments afterwards is very quick and the membrane of the semi-matured vesicles is dense. If the cosolvent and water is weakly repulsive, LLPS could be observed within a wide range of solvent composition, the evolution of vesicle is relatively slow, and the membrane is loose. Moreover, when the initial water fraction rises, micellization is preferential, and at a specific solvent composition, the use of cosolvent with strong affinity to water would promote to form small vesicles via an elongated semi-vesicle intermediate. With the further increase of water fraction, in both cases, only bilayer bending and closing processes are observed. It is found that the adsorption of the cosolvents in the polymer domains and the resulting hydrophilic chain conformations are critical for the control over the vesicle formation pathway. Our findings would be helpful for molecular engineering of the vesicle structures and for further potential applications. It also should be mentioned, the vesicular structures have been observed within a broad range of hydrophilic content of copolymers, and in experiments the choice of solvents would be subtle on the control over the final morphologies. Therefore, it is worthwhile to investigate the self-assembly of copolymers with the varied hydrophilic-to-hydrophobic balance and to explore the delicate influences of solvents on the formation and mechanisms of different morphologies in future.

## Figures and Tables

**Table 1:** Interaction parameters *a_ij_* in $F_{ij}^{C}$ used in the present work.

	W	G	A	B
W	25			
G	15, 30	25		
A	25	25	25	
B	50	25	50	25

## Data Availability

The data presented in this study are available on request from the corresponding author.

## Supplementary Materials

The following supporting information can be downloaded at: https://www.mdpi.com/article/10.3390/molecules28135113/s1, Figure S1: The self-assembly of A_2_B_12_ during solvent exchange at *ϕ*_W_ = 40% in the case of *a*_WG_ = 15. Figure S2: (a) The snapshot of an aggregate having *n*_agg_ = 32 observed at *ϕ*_W_ = 35% in the case of *a*_WG_ = 30. (b) Density profiles of water, cosolvent and copolymer blocks versus the distance from the mass center of the corresponding morphologies in (a). Figure S3: The self-assembly of A_2_B_12_ during solvent exchange in the case of *a*_WG_ = 30 starting from $\phi_{W}^{\mathrm{init}}$ = 60%. An equilibrium duration of 2.0 × 10^6^ time steps at $\phi_{W}^{\mathrm{init}}$ = 60% and an exchange frequency of *t*_eq_ = 2.0 × 10^5^ time steps are adopted.
