# Supplementary material for: Effect of Cosolvent on the Vesicle Formation Pathways under Solvent Exchange Process: A Dissipative Particle Dynamics Simulation
Source: Molecules. 2023 Jun 29;28(13):5113. doi: 10.3390/molecules28135113 (PMC10343840; doi:10.3390/molecules28135113)
Supplement: Supplementary file 1 [file molecules-28-05113-s001.zip › molecules-2464232-supplementary.pdf]

Supporting Information

Effect of Cosolvent on the Vesicle Formation Pathways under Solvent Exchange Process: A Dissipative Particle Dynamics Simulation

Zhonglin Luo *, Zhou Shu, Yi Jiang and Biaobing Wang *

Jiangsu Key Laboratory of Environmentally Friendly Polymeric Materials, Jiangsu Collaborative Innovation Center of Photovolatic Science and Engineering, National Experimental Demonstration Center for Materials Science and Engineering, School of Materials Science and Engineering, Changzhou University,
Changzhou 213164, China; zhoushu202306@163.com (Z.S.); yijiang202306@163.com (Y.J.)
* Correspondence: zhonglinluo@cczu.edu.cn (Z.L.); biaobing@cczu.edu.cn (B.W.)

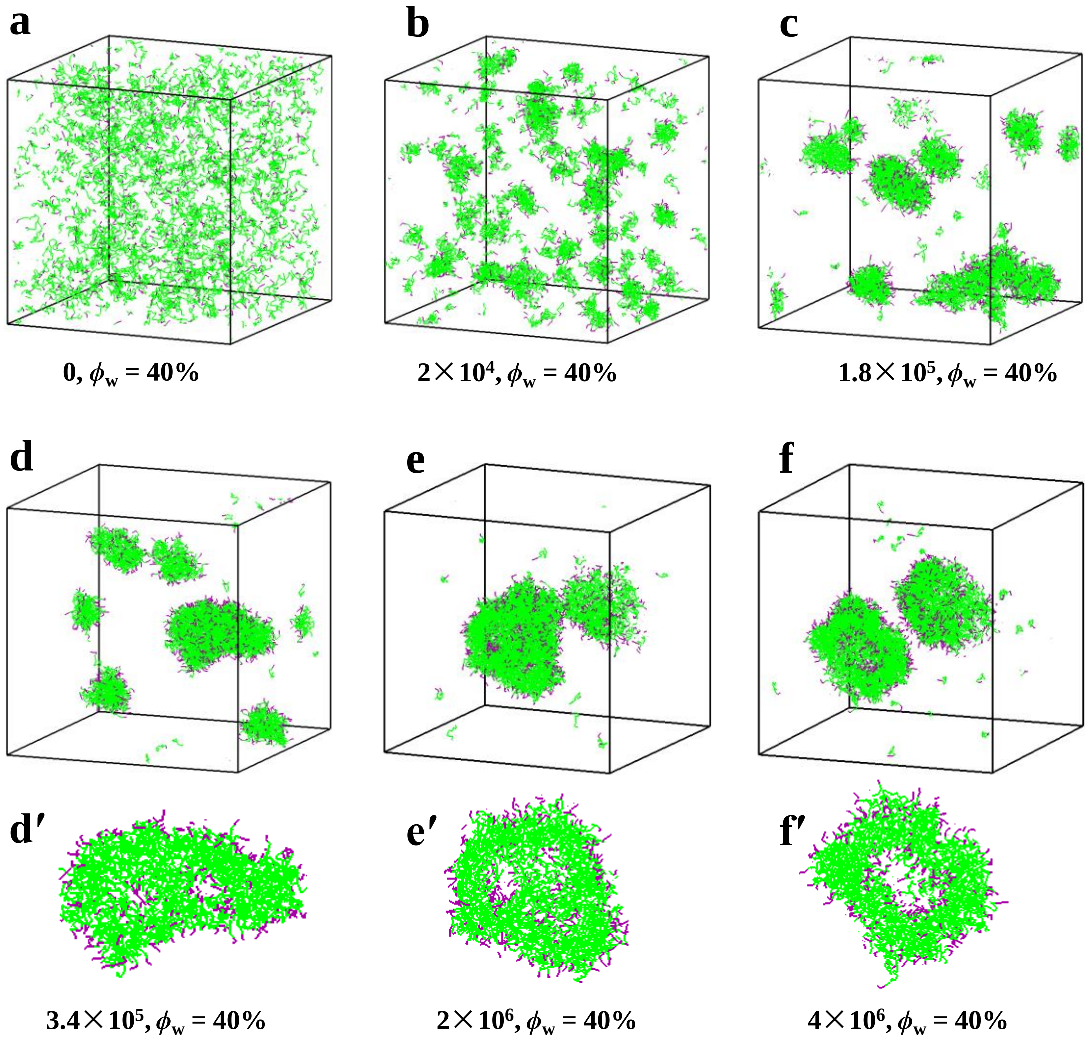

Figure S1. The self-assembly of A_2B_{12} at ϕ_W = 40% in the case of a_{WG} = 15.

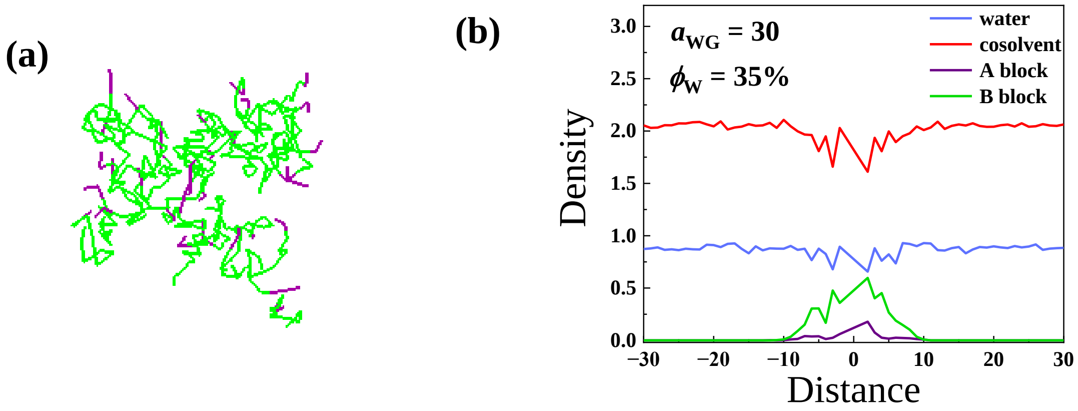

Figure S2. (a) The snapshot of an aggregate having n_{agg} = 32 observed at ϕ_W = 35% in the case of a_{WG} = 30. (b) Density profiles of water, cosolvent and copolymer blocks versus the distance from the mass center of the corresponding morphologies in (a).

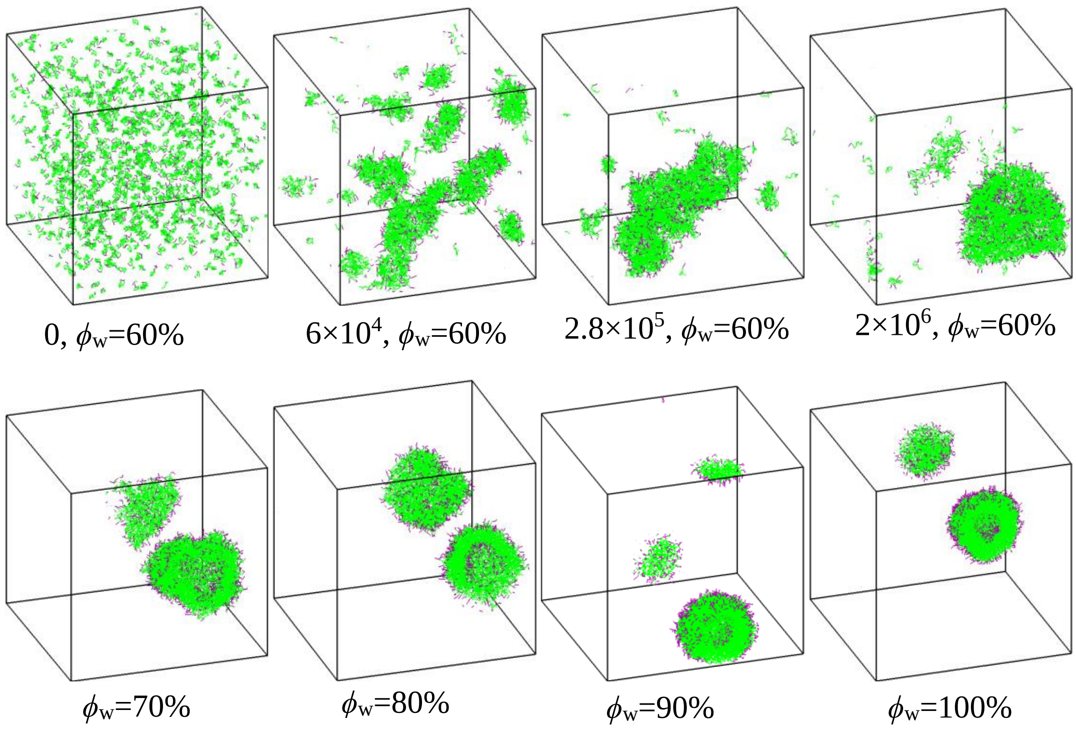

Figure S3. The self-assembly of A_2B_{12} during solvent exchange in the case of a_{WG} = 30 starting from ϕ_W^{init} = 60%. An equilibrium duration of 2.0×10^6 time steps at ϕ_W^{init} = 60% and an exchange frequency of t_{eq}= 2.0×10^5 time steps are adopted.
